# Erosion and sedimentation during the September 2015 flooding of the Kinu River, central Japan

**DOI:** 10.1038/srep34168

**Published:** 2016-09-28

**Authors:** Dan Matsumoto, Yuki Sawai, Masaki Yamada, Yuichi Namegaya, Tetsuya Shinozaki, Daisuke Takeda, Shigehiro Fujino, Koichiro Tanigawa, Atsunori Nakamura, Jessica E. Pilarczyk

**Affiliations:** 1 Research Institute of Earthquake and Volcano Geology, National Institute of Advanced Industrial Science and Technology (AIST), Tsukuba Central 7, 1-1-1 Higashi, Tsukuba, Ibaraki 305-8567, Japan; 2 Graduate School of Life and Environmental Sciences, University of Tsukuba, 1-1-1 Tennodai, Tsukuba, Ibaraki 305-8572, Japan; 3 Faculty of Life and Environmental Sciences, University of Tsukuba, 1-1-1 Tennodai, Tsukuba, Ibaraki 305-8572, Japan; 4 Division of Marine Science, University of Southern Mississippi, Stennis Space Center, Mississippi, 39529, USA

## Abstract

Erosional and sedimentary features associated with flooding have been documented in both modern and past cases. However, only a few studies have demonstrated the relationship between these features and the corresponding hydraulic conditions that produced them, making it difficult to evaluate the magnitude of paleo-flooding. This study describes the characteristics associated with inundation depth and flow direction, as well as the erosional and sedimentary features resulting from the disastrous flooding of the Kinu River, central Japan, in September 2015. Water levels rose rapidly due to heavy rainfall that eventually overtopped, and subsequently breached, a levee in Joso City, causing destructive flooding on the surrounding floodplain. Distinctive erosional features are found next to the breached levee, while depositional features, such as a sandy crevasse-splay deposit are found further away from the breach. The deposit can be divided into three units based on sedimentary facies. The vertical and lateral changes of these sedimentary facies may be the result of temporal and spatial changes associated with flow during the single flooding event. These observations and quantitative data provide information that can be used to reveal the paleohydrology of flood deposits in the stratigraphic record, leading to improved mitigation of future flooding disasters.

The disastrous flooding along the Kinu River in September 2015 was the result of intensive rainfall in central Japan. Fluvial flooding is among the most destructive natural disasters, resulting in significant damages to densely populated areas located in or near low-lying river basins^1^. Reconstructions of paleoflood hydrology are needed to improve prevention strategies to mitigate future flooding events. The accuracy of such reconstructions is enhanced by the detailed studies of modern analogues^2^.

This study documents the patterns of erosional scour and sedimentary deposition that occurred on the floodplain during the September 2015 flooding of the Kinu River in central Japan. Erosional features and sedimentary deposits associated with modern^3^,^4^,^5^ and past^6^,^7^,^8^ floods were investigated in previous studies. However, the link between their formation and hydraulic conditions was not specifically addressed. To investigate this link, we surveyed the inundation depth and flow direction shortly after the flood waters receded, and documented the erosional and depositional features at neighboring stations. We conducted grain-size and diatom analyses in the laboratory to examine the depositional processes in relation to the hydrologic conditions associated with the flooding.

## The Kinu River and the September 2015 flooding

**Figure 1:**
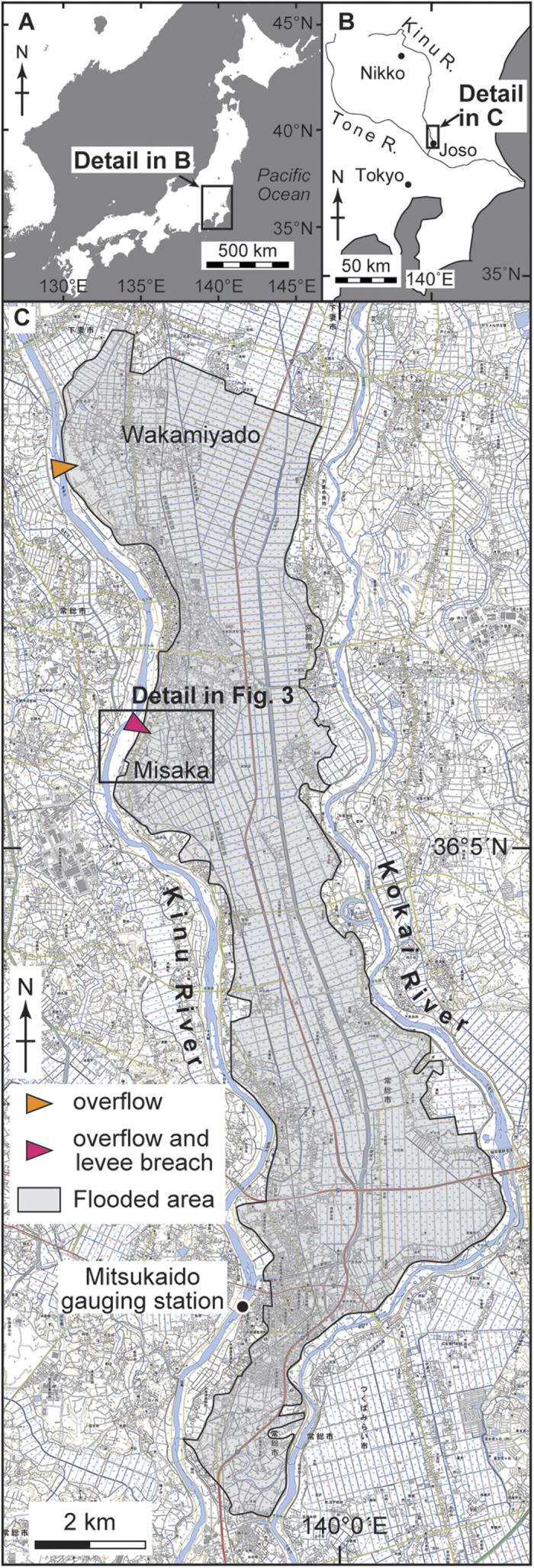
Location map of the study area. (**A**) General map of Japan. (**B**) Map of the Kinu River in central Japan. (**C**) Index map around the study area in Joso City showing the flooded area, position of the Mitsukaido gauging station, and the overflow and levee-breach points along the Kinu River during the 2015 flooding. GMT 4.5.6 software^35^ was used to prepare the maps in panels (**A**) and (**B**). A Digital Topographic Map 25000 from the Geospatial Information Authority of Japan^36^ was used as the basemap in panel (**C**).

The Kinu River in central Japan is the main tributary of the Tone River which discharges into the Pacific Ocean (Fig. 1A,B). The Kinu River originates just north of Nikko City, flows for 180 km in a north-south direction (Fig. 1B), and has a slope gradient of 0.05–0.10% in its downstream area^9^. The river often overflows because it drains a large and steep basin and its lower section is characterized by a channel meandering in a particularly flat plain. Many flooding disasters were recorded along the Kinu River and the neighboring Kokai River (Fig. 1C)^10^, and several have occurred in the vicinity of the study area since 1868 (see Supplementary Information). For example, the Kinu River breached the levee and generated a large-scale inundation near the study area during the 1949 flood.

**Figure 2:**
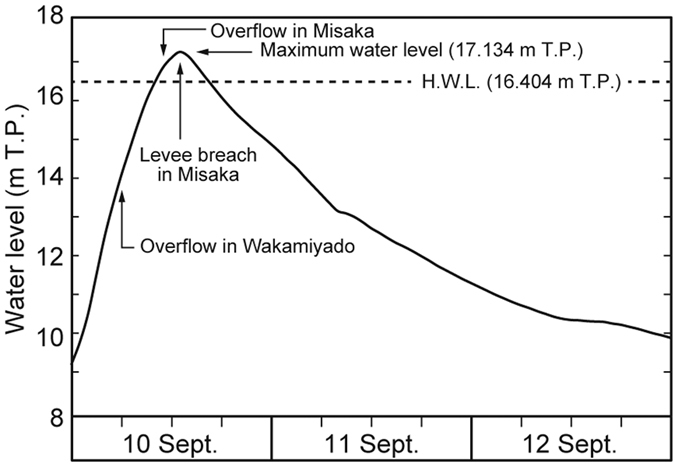
Hydrograph for the Kinu River at the Mitsukaido gauging station (Fig. 1B) from 10 to 12 September 2015. Measured water levels^37^ were expressed compared to Tokyo Peil (T. P.; mean sea level in Tokyo Bay). H.W.L. means High Water Level at this station, a threshold height for flooding relative to the neighboring levee height.

**Figure 3:**
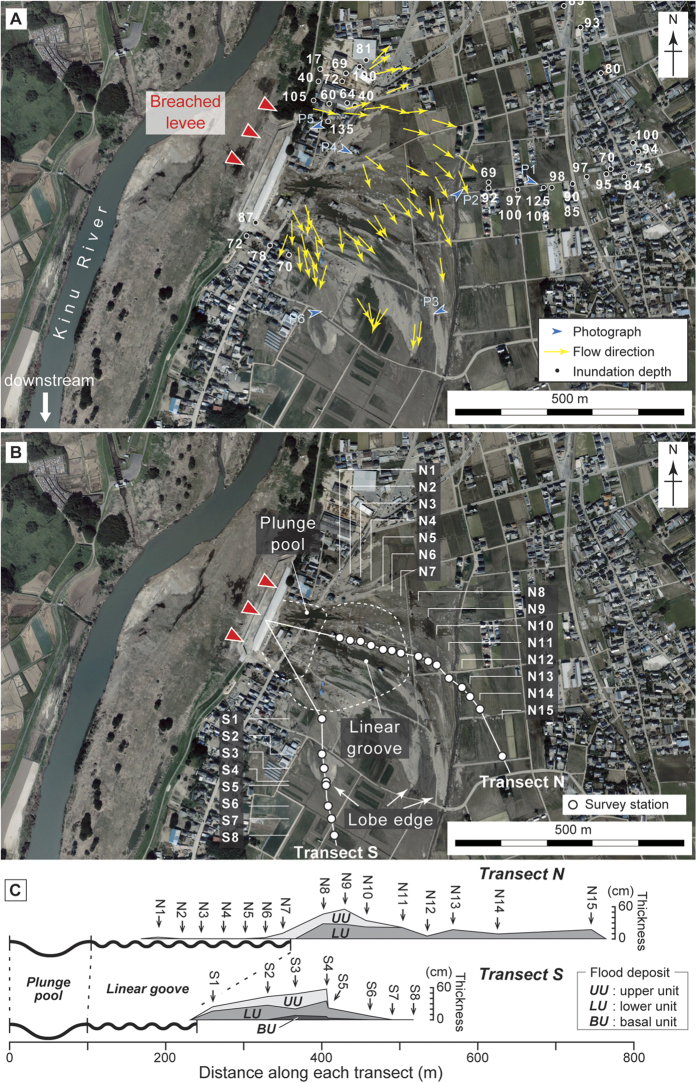
Detailed map of the survey sites with panels A and B displaying the same area. The aerial photo was obtained from the Geospatial Information Authority of Japan^38^ (CKT-2015-12-C4-14) and taken on 29 September, 2015. (**A**) Map showing the survey locations on the photograph (blue arrowhead; Fig. 4), flow direction (yellow arrow) and inundation depth (black circle with the measured depth). (**B**) Map showing the survey stations where erosional and depositional features along Transects N and S were investigated. Transects N and S start at the breached levee and extend in the direction of flow. (**C**) Sectional view of the lateral distributions of erosional and sedimentary features along each transect from the breached levee. Note that erosional depths are not to scale.

Heavy rainfall triggered by a tropical storm occurred on 9–11 September 2015, leading to catastrophic river flooding around Joso City, central Japan (Fig. 1B,C). A meteorological station (Imaichi Station) located in central Nikko City (Fig. 1B) recorded 366.5 mm/day of rain on 9 September, which is the most intense rainfall event recorded at the station since 1978^11^. Water levels in the Kinu River rapidly rose on 10 September as a result of the rainfall. In the downstream area of the Kinu River, the Mitsukaido gauging station in Joso City (Fig. 1C) recorded a maximum discharge of 4000 m^3^/s, which is the largest amount recorded at that location since 1936^12^. At the station, water levels rose by 8 m over a 12 hour interval, and reached a maximum height of 17.134 m T.P. (Tokyo Peil; mean sea level of Tokyo Bay) at 10 a.m. on 10 September (Fig. 2), which exceeded the high water level (a threshold height for flooding relative to the neighboring levee height; 16.404 m T.P.). As a result, the Kinu River began to overtop its left bank at two locations in Joso City area (colored arrows in Fig. 1C) on 10 September. Flood waters overtopped the levees in Wakamiyado (orange arrow in Fig. 1C) at 6 a.m. and in Misaka (red arrow in Fig. 1C) at 11 a.m. Finally at 1 p.m., the 200-meter wide levee in Misaka was completely breached resulting in disastrous flooding^9^. Based on video analysis, the inundation flow velocity measured just behind the breached levee reached up to 4.5 m/s^13^. All the floodplain located between the Kinu and Kokai Rivers was inundated and the flood affected a 40 km^2^ area around Joso City^14^ (Fig. 1C). The study area is located near the breached levee in the Misaka area in Joso City (Figs 1C and 3).

## Results

**Figure 4:**
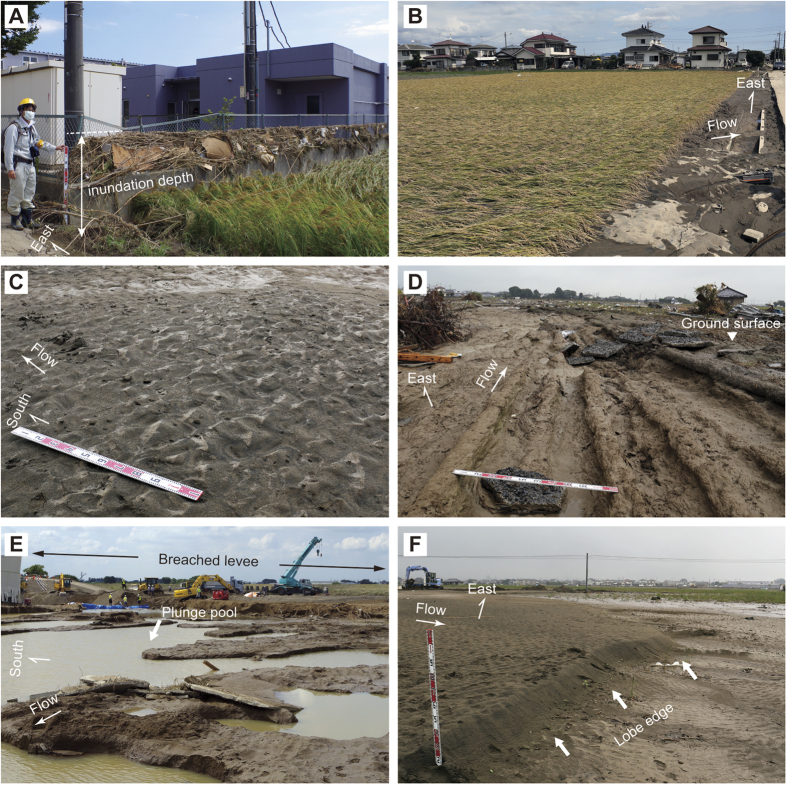
Photographs of the study area. Locations are shown in Fig. 3A (P1–6). The scale bar in panels A, C, D and F represents 1 m. (**A**) Water-borne debris on the fence at P1 showing the inundation depth of flooding. (**B**) Leaning rice plants showing the direction of flooding water at P2. (**C**) Ripple marks on the surface of the flood deposit indicating the flow direction at P3. (**D**) Linear grooves along the flow direction at P4. (**E**) Plunge pools scoured by the 2015 flood just behind the breached levee at P5. (**F**) Lobe edge of the flood deposit at P6.

The water depth during flooding, deduced from water marks and water-borne debris (Fig. 4A), was generally constant at most of the surveyed locations within the study area and ranged from 17 to 135 cm (83 cm on average) (Fig. 3A). Leaning plants, bedforms, and erosional scours (Fig. 4B–D) indicated an ESE-SSE flow direction that was perpendicular to the Kinu River flow in the area next to the breached levee (Fig. 3A). With increasing distance away from the breached levee, the direction of the flow gradually turned southward (Fig. 3A).

**Figure 5:**
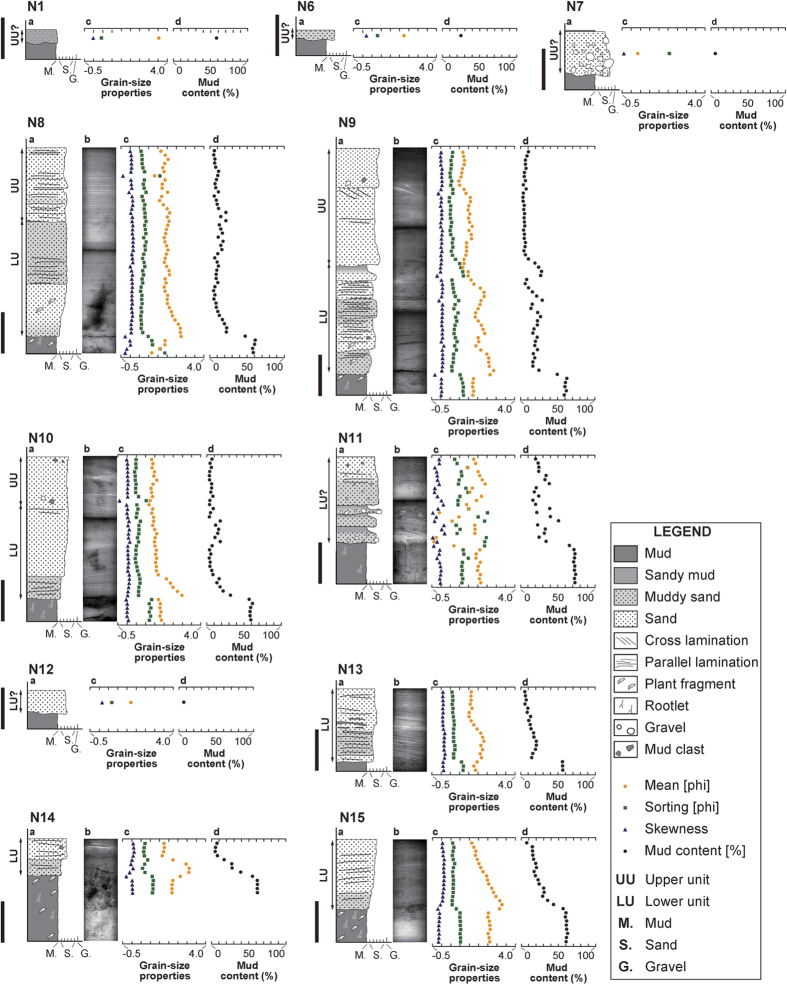
Schematic column (**a**), soft X-ray image (**b**), graph of grain-size properties (mean, sorting and skewness) of the particles larger than 63 μm (**c**), and mud content (**d**) of the flood deposit along Transect N (N1, N6–15). The scale bar beside each column represents 10 cm.

**Figure 6:**
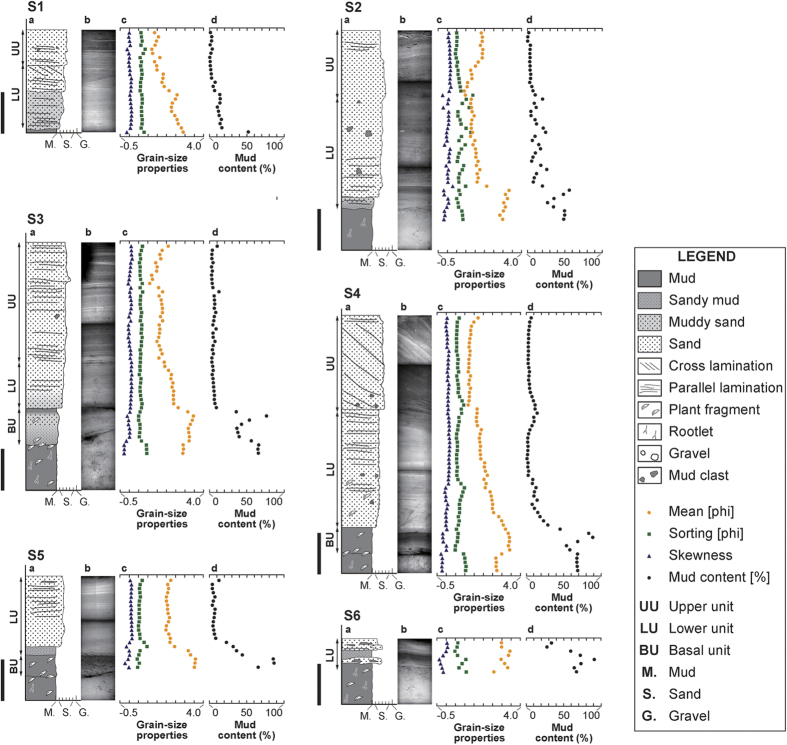
Schematic column (**a**), soft X-ray image (**b**), graph of grain-size properties (mean, sorting and skewness) of the particles larger than 63 μm (**c**), and mud content (**d**) of the flood deposit along Transect S (S1–6). The scale bar beside each column represents 10 cm.

Numerous geomorphological features of erosion and sediment deposition resulting from the flooding were found within the study area. These features, including plunge pools, linear grooves and flood deposits, were exclusively found in a small area located next to the breached levee and were parallel to the main flow direction. Plunge pools, scoured by the flooding water, were several meters deep, 100 m wide and found at the toe of the breached levee’s back slope (at P5 in Figs 3A,C and 4E). Roads and houses close to the plunge pools were completely destroyed and swept away. Adjacent to the plunge pools, numerous well-developed linear grooves were observed (Fig. 4D). The linear grooves were orthogonal to the Kinu River and found in a zone that extended for 250 m along the direction of flow. These grooves were observed at stations N1–7 along Transect N, and between the plunge pools and station S1 along Transect S (Fig. 3B,C). In the linear groove zone, up to 1 m of the pre-flood soil was laterally eroded, causing the underlying semi-consolidated muddy floodplain deposits to be exposed. Linear grooves that were several tens of centimeters deep and wide were dug further into the floodplain deposits as scour marks. The erosional surface was covered by either a thin mud drape (generally less than 1 cm thick; at N2–5; Fig. 3B) or by a thicker sandy flood deposit (generally several centimeters thick, with a maximum depth of 10 cm; at N1, N6–7; Figs 3B and 5). The flood deposit was limited to the area surrounding N1, N6–15 and S1–6 (Figs 3B,C, 5 and 6). In some places (N2–5, S7–8), only a thin mud drape overlay the surface. Bedforms such as unidirectional current ripples and dunes were widely found at the surface within the study area (Fig. 4C). At the midpoint between S4 and S5, the bedforms comprised a stepwise architecture resembling a deltaic lobe ranging heights comprised between 20–30 cm (Figs 3B,C and 4F).

The 2015 flood deposit overlay soil or semi-consolidated floodplain deposits, and the profile was characterized by a clear erosional boundary. The flood deposits were easy to distinguish because of their unique sedimentary facies and the abundance of fallen plants just below them. Grain-size analysis revealed that on average, the flood deposit was mainly composed of moderately-sorted, fine-skewed, medium sand (1.69 phi in mean grain size, 0.94 phi in sorting, and 0.13 in skewness) with a mud content of 15.19%. The underlying soil was a poorly-sorted, coarse-skewed, fine sand (2.02 phi in mean grain size, 1.19 phi in sorting, and −0.13 in skewness) with a much higher mud content of 62.55% (Figs 5 and 6). Soft X-ray radiography indicated the presence of mud clasts, gravels, and parallel and cross laminations at various depths in the flood deposits. (Figs 5 and 6).

The latter could be divided into three sedimentary units based on depositional features and grain-size. The basal unit consists of a muddy deposit, while the lower and upper units consist of sandy material. The basal unit contained abundant plant fragments and a higher mud content compared to the other units at S3–5 (Figs 3B and 6). In general the lower and basal units were characterized by distinct inverse grading although normal grading on a smaller scale was observed in some places. (Figs 5 and 6) Parallel laminations with a slightly higher mud content were present with an upward-decreasing trend within the lower unit. At some stations, the lower unit contained small-scale cross laminations, mud clasts and gravel grains. By contrast, the upper unit generally consisted of coarser sand and a slight fining trend in grain size (Figs 5 and 6). Sedimentary structures in the upper unit were variable and include parallel laminations and distinct large-scale cross laminations which reached a thickness of 20 cm at station S4 (Figs 3B and 6). Mud clasts, gravel and a thin mud drape and/or surface ripple marks were also common (Fig. 4C).

Sedimentary and erosional features found in the flood deposits were laterally variable across the study area (Fig. 3C). The basal unit of the flood deposit was only found at three stations along Transect S. The lower unit was generally confined to areas distant from the breached levee, whereas the upper unit was generally confined to areas close to the breached levee. The lower unit of the flood deposit was found at stations N8–15 and S1–8, while the upper unit was found at stations N1, N6–10, and S1–4 (Figs 3B, 5 and 6). The latter overlapped the former at N8–10 and S1–4 where they showed a vertical stacking pattern with lateral offset. Abrupt lateral changes in thickness and sedimentary features were occasionally found in the two units. With increasing distance from the Kinu River, the lower unit showed a slight thinning trend, while the upper unit showed a slight thickening trend along both transects (Fig. 3C). Along Transect N, the lower unit thinned from 28 cm at N8 to 17 cm at N15, while the upper unit thickened from 3 cm at N1 to 12 cm at N10. Along Transect S, the lower unit thinned from 16 cm at S1 to 7 cm at S6, while the upper unit thickened from 8 cm at S1 to 22 cm at S6. At stations N9 and N11, the lower unit was characterized by a series of graded layers, although more commonly, a single inversely-graded layer was present (Fig. 5). In general, the upper unit consisted of a sandy graded layer. However, at stations N1 and N6, the upper unit consisted of either a thin muddy-sand or a sandy-mud layer and transitioned into gravelly sand at station N7 (Fig. 5).

**Figure 7:**
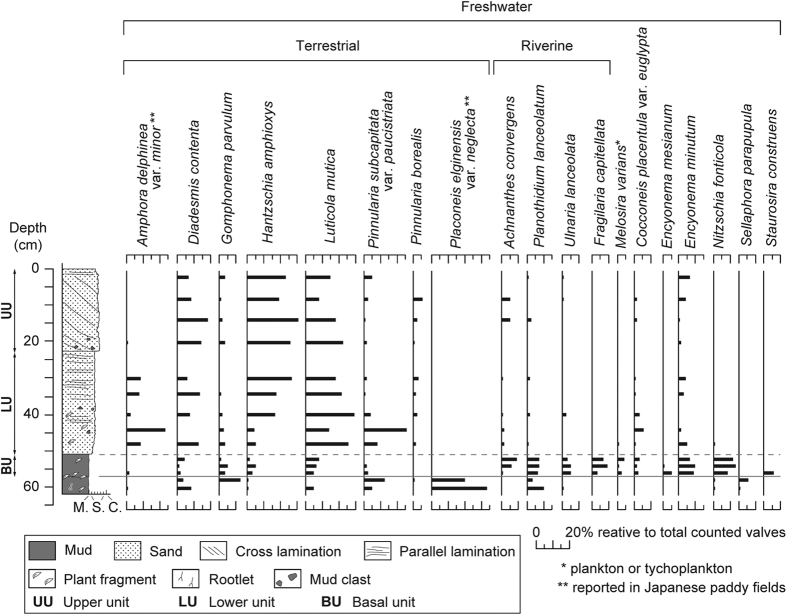
Diatom assemblages for the flood deposit at S4 showing changes in the relative abundance of the dominant species.

Diatom analysis for the deposit found at station S4 revealed that freshwater species were generally dominant, but the species composition of the assemblages varied as a function of the sedimentary facies (Fig. 7). The underlying soil contained many freshwater terrestrial taxa such as *Placoneis elginensis* var. *neglecta*, *Pinnularia subcapitata* var. *paucistriata*, and *Gomphonema parvulum*. These assemblages abruptly changed at the contact between the underlying soil and the muddy basal unit. The basal unit was characterized by riverine benthic diatom species (*Planothidium lanceolatum* and *Ulnaria lanceolata*)^15^, other freshwater species, and a decrease in terrestrial taxa (*Diadesmis contenta* and *Gomphonema parvulum*). In the sandy lower and upper units, diatom assemblages were dominated by terrestrial taxa (e.g. *Diadesmis contenta*, *Hantzschia amphioxys*, and *Luticola mutica*).

## Discussion and Conclusions

Flow direction deduced from leaning plants and sedimentary features provides a way to reconstruct the dispersing process of the inundation flow. By contrast, the inundation depth (Fig. 3A) corresponds to the maximum height of slackwater rather than the water depth flowing from the breached levee during the flooding. This is because the inundation depth was generally constant regardless the flow direction within the study area. Our field observations and laboratory analyses show that erosional and depositional processes associated with flood waters from the Kinu River varied in time and space. The plunge pools and linear grooves found near the breached levee are consistent with the occurrence of strong currents that inundated the floodplain when the Kinu River overflowed. For example, strong inundation flow resulting from the 2011 tsunami caused plunge pools to form at the back toe of seawalls^16^, which ultimately resulted in their failure^17^. Initial overflow from the Kinu River may have accelerated the development of these pools, leading to the subsequent levee breach. The linear grooves can be classified as gutter casts which are a type of scour mark that has been commonly documented in nearshore zones where erosion and the bypass of sediment are dominant^18^. Gutter casts were also found at locations inundated by the 2011 Tohoku tsunami^19^. Erosion is the most conspicuous effect of the Kinu River flooding and will likely be preserved in the stratigraphic record^20^. This result agrees with several other studies that document the presence of erosional features in association with modern^21^ and past^6^ flooding events.

Sedimentary features found in the three units of the flood deposits are generally different. The differences between the three units may be related to the changes in inundation flow over the duration of the flood. The basal unit, characterized by high mud content, can be interpreted as deposition resulting from suspended load during the early stage of flooding when overtopping flow was unable to transport coarser sediments beyond the levee. By contrast, the sandy lower unit is characterized by inversely-graded sediments that were deposited by an increasing supply of suspended- and/or bed-load during the waxing flood stage when the concentration of fine sediments had relatively decreased^22^. The vertical decreasing trend in mud content is consistent with the depositional processes. The inverse-grading trend that was characteristic of the lower unit is a common sedimentary feature in flood deposits^21^,^22^,^23^. This process usually occurs when there is a rapid rise in water level in association with heavy rainfall, and it rarely occurs in case of gradual rise, such as a snowmelt^22^. Deposition of the basal and lower units that contain these features can be explained by overflow from the Kinu River. The slight upward-fining trend observed in the upper unit may reflect sediment deposition during a waning stage, while the mud drape on the surface indicates deposition during a stagnant stage of the flooding event. The large-scale cross laminations in the upper unit at station S4 are the result of the prograding lobe which ceased somewhere between stations S4 and S5 (Figs 3B and 4F). Similar depositional patterns have been reported in association with other recent flood deposits^21^,^24^. These features suggest that the upper unit was deposited by a stronger flow after the levee was breached. Parallel laminations were common in both the lower and upper units at most stations (Figs 5 and 6), which indicates the occurrence of a strong current in the upper flow regime^25^. Mud clasts and gravels were also found scattered throughout the deposits at most of the stations and suggest occurrence of scouring and transport by a high-energy current.

Distinctive distributions of the erosional (except for small-scale erosional contacts between soil and flood deposit) and sedimentary features throughout the study area reflect the spatial variability of inundation flow. Flooding from the Kinu River was confined to a narrow area where flood water both overtopped and breached the levee, generating strong inundation flow. Near the levee, where inundation flow was the strongest, erosional features were found. As flood waters flowed outwards from the levee, they diverged and decreased in strength, at which point sediment began to deposit on the pre-flood surface. The limited distribution of the basal unit along Transect S implies that the overtopping river waters, containing higher mud concentrations flowed southward along Transect S during the early stage of flooding. The observed lateral thinning trend in the lower unit can be explained by spatial waning of the flow due to a reduction in transport capacity. The lateral thickening trend in the upper unit was likely the result of the formation, progradation and eventual halt of the lobe, although the possibility still remains that part of the basal and lower units were eroded by the same flow that formed the upper unit. The flood deposit as a whole shows a thickening-thinning pattern across the foreset slope of the prograding lobe, a pattern that has been reported in association with flood deposits elsewhere^26^.

Erosional and depositional features documented in this study are similar to other modern and past flood deposits. Erosional features that are limited to the area near the breached levee correspond to crevasse channel geometry^26^. Based on diagnostic sedimentary characteristics, the basal and lower units can be interpreted as deposition resulting from flow that overtopped the levee, while the upper unit can be interpreted as deposition resulting from the breached levee^21^,^22^. These erosional and sedimentary features were found within a relatively narrow area near the breached levee, even though the flood impacted a much greater area. Other studies have documented geomorphological features associated with past flooding using a combination of geophysical techniques such as ground-penetrating radar (GPR)^26^,^27^ and detailed geological surveys.

Distinct changes in diatom assemblages are recognized within the deposit at S4. The muddy basal unit of the flood deposit is characterized by a mixture of benthic, planktonic riverine and terrestrial freshwater diatom species. These assemblages were likely to be formed from suspended load due to the initial overtopping of the levee. Because diatom valves are known to exhibit the same depositional behavior as that of silt-sized sediment grains, the dominant species were probably incorporated with the fine fractions of the deposit. The sandy lower and upper units of the flood deposit were dominated by terrestrial diatom species (*Diadesmis contenta*, *Hantzschia amphioxys*, and *Luticola mutica*). Such species are commonly found on the surface of wet soils and mosses, but not in coarse-grained sediments^28^,^29^,^30^. This implies that living diatoms were transported from their original habitat, or preexisting valves that were buried in soils were scoured, and incorporated with flood sediments during high-energy inundation flow. The observed difference in assemblages can be attributed to the different origins and transportation processes, which are consistent with the depositional processes deduced from the sedimentary features.

In summary, this study documents the features of erosion and sediment deposition associated with the September 2015 flooding of the Kinu River. The inundation depth, flow direction, and dynamics associated with the formation of sedimentary features during the flood are also described. The observed erosional and depositional characteristics indicate that two different processes were involved during the single flooding event. The detailed descriptions of sedimentary characteristics associated with this modern flooding event reveal distinct features that can be used to better understand the history of past fluvial and flooding events. Furthermore, results from this study establish a link between sedimentary and erosional features and the mechanisms that formed them during the flood. This link is important to understand why flood deposits worldwide are variable and do not always show the same features. For example, some erosional and sedimentary features documented in this study are similar to those found in association with modern flood deposits elsewhere in Japan^5^,^22^,^23^ as well as in other countries^3^,^21^. However, these features are generally dissimilar to those described in association with the extraordinary 2011 Mississippi River flood^31^,^32^. The 2011 Mississippi deposit is characterized by a laterally extensive thin muddy deposit that overlies a flat area influenced by subsidence. The deposit contains diatom assemblages characterized by abundant riverine taxa, similar to those found in the basal unit in the current research. The flood deposits might vary their features corresponding to types of flooding and surrounding environments. An improved understanding of the flooding history provides useful information for future disaster mitigation.

## Methods

Field investigations were conducted around the Misaka area in Joso City on 14–16 and 25 September 2015. At 34 locations, we used leveling rods to measure the inundation depth of the flooding using water marks and water-borne debris preserved on walls and fences. Flow direction was inferred from leaning plants and bedforms at 54 locations. Investigations of erosional and depositional features were done at 23 stations along the two transects; Transect N and Transect S. Both transects extended from the breached levee following in the direction of flow. We conducted observation of the flood deposits at 15 and 8 survey stations along Transect N (650-meter long) and Transect S (500-meter long) respectively. Using a handy geoslicer, we collected columnar sediment samples of the flood deposit at 13 stations (N8–11, N13–15, S1–6) along the two transects, and single bulk samples at 4 stations (N1, N6–7, N12). Samples were not collected at six stations (N2–5, S7–8) because there was no discernable flood deposit, except a thin mud drape.

In the laboratory, all samples were photographed by soft X-ray after visual observation and subsampled at 1 cm intervals. All subsamples and bulk samples were sieved at 63 μm (4 phi) to remove the mud component, and analyzed for grain-size distributions for the sand fraction by using an image analyzer (Camsizer, Retsch Technology). A measuring range was set from −5.25 (pebble) to 6.25 (silt) at intervals of 0.25 in phi scale. Following analysis, the mean, sorting and skewness parameters of the grain-size distributions were calculated by using the logarithmic graphical method^33^.

Analysis of diatom assemblages was conducted on the pretreated samples^34^. Approximately 200 diatom valves were identified and counted from prepared slides from 14 horizons within the deposit at S4. To interpret diatom assemblages, we classified the freshwater taxa as terrestrial (subaerial), riverine benthic, or undetermined. Ecological information associated with each assemblage was based on Japanese local floral studies^15^,^29^,^30^.

## Additional Information

**How to cite this article**: Matsumoto, D. *et al.* Erosion and sedimentation during the September 2015 flooding of the Kinu River, central Japan. *Sci. Rep.* **6**, 34168; doi: 10.1038/srep34168 (2016).

## Supplementary Material

Supplementary Information
