# Supplementary material for: Erosion and sedimentation during the September 2015 flooding of the Kinu River, central Japan
Source: Sci Rep. 2016 Sep 28;6:34168. doi: 10.1038/srep34168 (PMC5039755; doi:10.1038/srep34168)
Supplement: Supplementary Information [file srep34168-s1.pdf]

Supplementary Information

Erosion and sedimentation during the September 2015 flooding of the Kinu River, central Japan

Dan Matsumoto[1]*, Yuki Sawai[1], Masaki Yamada[2], Yuichi Namegaya[1], Tetsuya Shinozaki[2]**, Daisuke Takeda[2], Shigehiro Fujino[3], Koichiro Tanigawa[1], Atsunori Nakamura[1] and Jessica E. Pilarczyk[4]

[1]*Research Institute of Earthquake and Volcano Geology, National Institute of Advanced Industrial Science and Technology (AIST), Tsukuba Central 7, 1-1-1 Higashi, Tsukuba, Ibaraki 305-8567, Japan*

[2]*Graduate School of Life and Environmental Sciences, University of Tsukuba, 1-1-1 Tennodai, Tsukuba, Ibaraki 305-8572, Japan*

[3]*Faculty of Life and Environmental Sciences, University of Tsukuba, 1-1-1 Tennodai, Tsukuba, Ibaraki 305-8572, Japan*

[4]*Division of Marine Science, University of Southern Mississippi, Stennis Space Center, Mississippi, 39529, USA*

* Correspondence to dan-matsumoto@aist.go.jp

** *Present Address: Center for Research in Isotopes and Environmental Dynamics, University of Tsukuba, 1-1-1 Tennodai, Tsukuba, Ibaraki 305-8577, Japan*

Supplementary Table S1. History of flooding events around the study area.

History of flooding events along the Kinu and Kokai Rivers around the study area (Ishige and Mitsukaido areas) from 1868 to 1981 based on two chronicles. Ishige Town is the north part, and Mitsukaido City is the south part of Joso City. Citations for excerpts from each chronicle are shown in square brackets. All local places indicated in each excerpt are located in and around Joso City.

Date	Chronicle of Ishige Town*	Chronicle of Mitsukaido City**
Sep. 1869		Rise in water level (Kinu River) [p.478]
Jul. 1870		Large flood (Kinu River), water gate of irrigation canal was swept away at Ezure. [p. 478]
Jul. 1871	Rise in water level (Kinu River) [p.1107]	
Oct. 1882	Flood (Kokai River) [p.857]	
Sep. 1885	Large flood (Kinu and Kokai Rivers), levee breach near Oshizuna [p.857, 1109]	
Aug. 1890	Large flood (Kinu River) [p.857]	
Sep. 1896	Flood (Kokai River) at two places including Kitamonma [p.857]	
1897	Large flood (Kokai River) [p.857]	
Aug. 1901	Large flood along the basin (Kokai River) [p.857]	
Sep. 1902	Levee breach at the castle (Kinu River) [p.857]	Levee breach at the Mistukaido Castle and several houses were washed away at Hashimoto. [p. 484]
Aug. 1906	Flood at Kitamonma (Kokai River) [p.857]	
Aug. 1910	Large flood (Kinu and Kokai Rivers), levee breach at Katakaku, and overtopping at Awano, both in Nishitoyoda. Overtopping and levee breach in Kamitsuma resulted in the inundaion of farmland at Shitte, Shibui, Kirigase and Maegawara. Thirty and fifteen houses were inundated at Sakade and Uchimoriya, respectively. About 2 km^2 of farmland was inundated at Uchimoriya, and 0.5km^2 at Nakashinden in Tobanoe. [p.857–859, 1111]	Large flood (Kinu and Kokai Rivers) [p. 486]
Sep. 1927	Large flood (Kokai River), levee breach at Nagaoshi [p.857, 1109]	
Sep. 1935	Large flood (Kokai River), levee breach at Ueno, Murata, and Takasu resulted in the inundaiton of about 120 km^2 of farmland. [p. 857, 860]	
Jun.–Jul. 1938	Large flood (Kinu and Kokai Rivers), 70 m of levee breach at Mitsukaido. Levee breach resulted in the inundation of 352 houses at Toyoda. About 3.2 and 1.9 km^2 of farmlands were inudated at Ishige and Toyoda, respectively. Artificial levee break for drainage along the Kinu River. [p. 857, 860–862, 1115]	Levee breach (Kokai River) at Toyoda. Houses were inundated above floor level at Mitsukaido, Ohno, Goka, and Mitsuma. [p. 492]
Sep. 1938	Flood (Kokai River), about 170 houses were inundated at Ishige. [p.857, 862]	
Jul. 1941	Large flood (Kinu and Kokai Rivers), levee breach near Mukoishige, and overflow at Mitsuma. [p. 857, 1115]	Flood (Kinu River) resulted in the inundation below floor level at the lowland on the left side of the Kinu River. [p. 492]
1943	Flood (Kokai River) [p. 857]	
Sep. 1949	Flood (Kinu River), levee breach near Juichimen resulted in the inudation at Nishihara, Kamiishige, and Motoishige. [p. 1117]	
Jun. 1961		Inundation above floor level due to heavy rain. Roads, bridges and levees were broken. [p. 498]
Sep. 1971	Rise in water level (Kokai River), levee breach at the north of Shiinoki in Toyoda [p.1122]	
Aug. 1981	Flood water inundated houses below floor level, and damaged on the harvest. [p. 1124]	

References

* Compilation Committee for Chronicle of Ishige Town (*ed.*) Chronicle of Ishige Town (Ishige Chosi), pp. 1133. (in Japanese, original editor and title translated) (Seikosha Printing, 1988)

** Compilation Committee for Chronicle of Mitsukaido City (*ed.*) Chronicle of Mitsukaido City Volume 2 (Mitsukaido Shishi Gekan), pp. 503. (in Japanese, original editor and title translated) (Gyosei Corp., 1985)
